# Associations of Plasma Phospho-Tau217 Levels With Tau Positron Emission Tomography in Early Alzheimer Disease

**DOI:** 10.1001/jamaneurol.2020.4201

**Published:** 2020-11-09

**Authors:** Shorena Janelidze, David Berron, Ruben Smith, Olof Strandberg, Nicholas K. Proctor, Jeffrey L. Dage, Erik Stomrud, Sebastian Palmqvist, Niklas Mattsson-Carlgren, Oskar Hansson

**Affiliations:** 1 Clinical Memory Research Unit, Department of Clinical Sciences Malmö, Lund University, Sölvegatan, Sweden; 2 Department of Neurology, Skåne University Hospital, Lund, Sweden; 3 Eli Lilly and Company, Indianapolis, Indiana; 4 Memory Clinic, Skåne University Hospital, Malmö, Sweden; 5 Wallenberg Center for Molecular Medicine, Lund University, Lund, Sweden

## Abstract

**Importance:**

There is an urgent need for inexpensive and minimally invasive blood biomarkers for Alzheimer disease (AD) that could be used to detect early disease changes.

**Objective:**

To assess how early in the course of AD plasma levels of tau phosphorylated at threonine 217 (P-tau217) start to change compared with levels of established cerebrospinal fluid (CSF) and positron emission tomography (PET) biomarkers of AD pathology.

**Design, Setting, and Participants:**

This cohort study included cognitively healthy control individuals (n = 225) and participants with subjective cognitive decline (n = 89) or mild cognitive impairment (n = 176) from the BioFINDER-2 study. Participants were enrolled at 2 different hospitals in Sweden from January 2017 to October 2019. All study participants underwent plasma P-tau217 assessments and tau- and amyloid-β (Aβ)–PET imaging. A subcohort of 111 participants had 2 or 3 tau-PET scans.

**Main Outcomes and Measures:**

Changes in plasma P-tau217 levels in preclinical and prodromal AD compared with changes in CSF P-tau217 and PET measures.

**Results:**

Of 490 participants, 251 were women (51.2%) and the mean (SD) age was 65.9 (13.1) years. Plasma P-tau217 levels were increased in cognitively unimpaired participants with abnormal Aβ-PET but normal tau-PET in the entorhinal cortex (Aβ-PET^+^/ tau-PET^–^ group vs Aβ-PET^–^/ tau-PET^–^ group: median, 2.2 pg/mL [interquartile range (IQR), 1.5-2.9 pg/mL] vs 0.7 pg/mL [IQR, 0.3-1.4 pg/mL]). Most cognitively unimpaired participants who were discordant for plasma P-tau217 and tau-PET were positive for plasma P-tau217 and negative for tau-PET (P-tau217^+^/tau-PET^–^: 36 [94.7%]; P-tau217^–^/tau-PET^+^: 2 [5.3%]). Event-based modeling of cross-sectional data predicted that in cognitively unimpaired participants and in those with mild cognitive impairment, both plasma and CSF P-tau217 would change before the tau-PET signal in the entorhinal cortex, followed by more widespread cortical tau-PET changes. When testing the association with global Aβ load in nonlinear spline models, both plasma and CSF P-tau217 were increased at lower Aβ-PET values compared with tau-PET measures. Among participants with normal baseline tau-PET, the rates of longitudinal increase in tau-PET in the entorhinal cortex were higher in those with abnormal plasma P-tau217 at baseline (median standardized uptake value ratio, 0.029 [IQR, –0.006 to 0.041] vs –0.001 [IQR, –0.021 to 0.020]; Mann-Whitney *U*, *P* = .02).

**Conclusions and Relevance:**

In this cohort study, plasma P-tau217 levels were increased during the early preclinical stages of AD when insoluble tau aggregates were not yet detectable by tau-PET. Plasma P-tau217 may hold promise as a biomarker for early AD brain pathology.

## Key Points

**Question:**

How early in the course of Alzheimer disease do plasma levels of tau phosphorylated at threonine 217 (P-tau217) start to change compared with levels of established cerebrospinal fluid and positron emission tomography (PET) tau biomarkers?

**Findings:**

In this cohort study of 490 individuals without dementia, plasma P-tau217 levels were elevated in amyloid-β–positive cognitively unimpaired participants before insoluble tau aggregates became detectable by tau-PET; modeling approaches predicted that both plasma and cerebrospinal fluid P-tau217 increased before tau-PET in the entorhinal cortex followed by more widespread cortical tau-PET changes.

**Meaning:**

The study results suggest that in Alzheimer disease, plasma P-tau217 becomes abnormal before tau-PET and that plasma P-tau217 may be considered as an early Alzheimer disease biomarker.

## Teaser

This cohort study compares plasma levels of phosphorylated tau at threonine 217 with established cerebrospinal fluid and positron emission tomography (PET) tau biomarkers in early Alzheimer disease.

## Introduction

In Alzheimer disease (AD), abnormal metabolism of amyloid-β (Aβ) and tau leads to accumulation of extracellular plaques formed by misfolded Aβ and intraneuronal neurofibrillary tangles containing phosphorylated tau (P-tau) protein.^1^ Neuropathological assessment of the amount and distribution of plaques and neurofibrillary tangles remains the criterion standard for AD diagnosis.^2^ Cerebrospinal fluid (CSF) analysis and positron emission tomography (PET) have also been widely used to detect and monitor AD-related amyloid and tau pathologies in individuals in research and clinical trials and, in some countries, in clinical practice.^3^ However, there is interest in developing blood tests for AD because blood sample collection is procedurally simple, minimally invasive, and time and cost-effective. Blood-based biomarkers are more suitable than CSF or PET for implementation in primary care settings worldwide and may reduce the costs of clinical trials by improving selection and stratification of participants and monitoring of treatment response.

Increasing evidence suggests that blood P-tau may be a useful diagnostic and prognostic biomarker of AD. Increased levels of plasma tau phosphorylated at threonine 181 (P-tau181) were initially reported in patients with AD dementia compared with cognitively unimpaired individuals.^4,5,6^ Two recent studies demonstrated that plasma P-tau181 accurately identified people with abnormal Aβ- and tau-PET scans and distinguished AD dementia from other non-AD neurodegenerative diseases including frontotemporal dementia.^7,8^ Furthermore, plasma P-tau181 predicted future progression to AD dementia with a high degree of accuracy in individuals without dementia.^7^ However, the tau protein has multiple phosphorylation sites, and although CSF P-tau181 has been extensively validated as a core biomarker of AD, emerging data indicate that CSF tau phosphorylated at threonine 217 (P-tau217) better reflects AD-related tau pathology.^9,10^ A recent study has suggested that plasma levels of P-tau217 start to change at the same time as CSF levels.^11^ In line with this, plasma P-tau217 has been shown to perform substantially better than plasma P-tau181 when differentiating patients with neuropathologically confirmed AD from those with no neuropathological evidence of AD.^12^ In the same study, plasma P-tau217 distinguished clinically diagnosed AD dementia from non-AD neurodegenerative disorders with accuracy comparable to CSF P-tau and tau-PET. In autosomal-dominant AD, plasma P-tau217 levels started to increase approximately 20 years before the estimated onset of mild cognitive impairment (MCI).

Although both CSF P-tau and tau-PET are considered as biomarkers of AD-related tau pathology, new findings suggest that they are not completely interchangeable. Cerebrospinal fluid levels of P-tau mirror changes in the brain tau metabolism and are elevated in all disease stages of AD, including the asymptomatic phase when tau-PET is still normal.^13,14,15^ Tau-PET tracers bind to insoluble paired helical filaments of tau in neurofibrillary tangles, and tau-PET measures become abnormal mainly in symptomatic AD and correlate with brain atrophy and cognitive function.^16,17^ Together, these findings suggest that fluid-based measurements of P-tau may be more sensitive than tau-PET in the earliest stages of AD. Although previous studies have found associations between plasma and CSF levels of P-tau,^7,8^ thus far it is unclear whether plasma P-tau could be used to detect early pathology in AD.

In the present study including a total of 490 participants, we examined the association between plasma P-tau217 and tau-PET focusing on early AD disease stages. We investigated associations between plasma P-tau217 and tau-PET signals in the entorhinal cortex (one of the earliest regions of AD-related tau pathology^18^) of cognitively unimpaired individuals. We also studied the order of change in plasma P-tau217, CSF P-tau217, and different tau-PET measures as well as associations between baseline plasma P-tau217 and longitudinal changes in entorhinal tau-PET in cognitively unimpaired participants and in those with MCI.

## Methods

### Study Participants

This cohort study included neurologically and cognitively healthy control individuals and participants with subjective cognitive decline or MCI from the prospective and longitudinal Swedish BioFINDER-2 study who underwent both tau-PET and Aβ-PET imaging. In accordance with the research framework by the National Institute on Aging and Alzheimer Association, study participants with subjective cognitive decline and cognitively healthy individuals were included in the group of cognitively unimpaired individuals.^3^ Of 505 eligible participants who underwent both tau- and Aβ-PET, 10 were excluded because scans did not meet the scan quality criteria; plasma samples were not available for another 2 individuals. Participants in the BioFINDER-2 study were recruited in southern Sweden (Skåne University Hospital and the Hospital of Ängelholm) between January 2017 and October 2019 as previously described^12^ (eMethods in the Supplement). The study was approved by the regional ethics committee in Lund, Sweden, and all participants gave written informed consent to participate.

### Plasma and CSF Sampling and Analysis

Blood samples were collected and handled as previously described.^7,19^ The procedure and analysis of CSF followed the Alzheimer Association flowchart for CSF biomarkers.^20^ Lumbar CSF samples were collected and analyzed according to a standardized protocol.^19,21^ Concentrations of plasma P-tau217^12^ and CSF P-tau217^10^ were measured using Meso Scale Discovery (Meso Scale Diagnostics)–based immunoassays at Lilly Research Laboratories by technicians who were blinded to the clinical and imaging data. For plasma analysis, biotinylated-IBA493 (biotin: Thermo Scientific; IBA493: Eli Lilly and Company) was used as a capture antibody and SULFO-TAG–4G10-E2 (anti-tau) (SULF-TAG: Meso Scale Diagnostics; 4G10-E2: Eli Lilly and Company) as the detector. For CSF analysis, biotinylated-IBA413 (Eli Lilly and Company) was used as a capture antibody and a tau-specific antibody (LRL; Eli Lilly and Company) as the detector. Plasma and CSF samples were diluted 1:2 and 1:8, respectively, in sample buffer containing heterophilic blocking reagent 1 at a concentration of 200 μg/mL (Scantibodies Inc). Plasma and CSF assays were calibrated using a recombinant tau (4R2N) protein that was phosphorylated in vitro using a reaction with glycogen synthase kinase 3 and characterized by mass spectrometry. Details of the assays are described in the eMethods in the Supplement. Plasma P-tau217 data were binarized (abnormal vs normal) using a predefined cutoff of 2.5 pg/mL. The cutoff was determined using the mean plus 2 SDs in a large group of Aβ-negative controls excluding 2 plasma P-tau217 outliers.^12^

### Tau- and Aβ-PET Imaging and Processing

Tau-PET, using RO948 F 18 ([^18^F]RO948).^22^ and Aβ-PET, using flutemetamol F 18 ([^18^F]flutemetamol), procedures are described in the eMethods in the Supplement. All assessments of imaging and clinical data were done blinded to plasma P-tau217 data.

### Statistical Analysis

Group differences in plasma P-tau217 levels were assessed with Mann-Whitney or univariate general linear models (log-transformed plasma P-tau217) adjusted for age and sex and least significant difference tests (post hoc) for pairwise group comparisons. Associations between plasma P-tau217, CSF P-tau217, tau-PET measures, and continuous Aβ-PET uptake were tested with nonlinear polynomial spline models (using I-spline basis) to estimate the change in biomarker levels by Aβ-PET load. To derive the sequence of biomarker abnormality, event-based modeling^23^ was used to compare 2 P-tau217 (plasma and CSF) and 3 tau-PET events (entorhinal, temporal meta, and neocortical meta-regions of interest [ROIs]) in which an event constituted a change toward biomarker abnormality (eMethods in the Supplement). To assess whether, similar to CSF P-tau217, plasma concentrations of P-tau217 become abnormally elevated before neurofibrillary tangles are detectable by tau-PET, we studied plasma P-tau217 levels in cognitively unimpaired participants in relation to Aβ-PET and tau-PET status. Tau-PET status was defined based on the [^18^F]RO948 standardized uptake value ratio (SUVR) in the entorhinal ROI and using a predefined cutoff of 1.48 SUVR.^22^ Simple mediation models were calculated using a bootstrap method for the mediated association.

For participants who had 2 or 3 tau-PET scans, yearly longitudinal changes in tau-PET SUVR were calculated as slopes from linear regression models with SUVR in the entorhinal ROI as dependent variable and time between the scans as a predictor.

Three outliers with plasma P-tau217 values 5 SDs above the mean of the whole cohort were excluded from the main analysis. The results including the outliers were similar. Of 490 study participants, 161 (32.9%) had plasma P-tau217 levels below the detection limit (0.48 pg/mL) of the assay. As previously shown, approximately 99% of individuals with plasma P-tau217 values below the lower detection are tau-PET^–^.^12^ All plasma P-tau217 values below the lower detection limit were included in the main part of this study. Plasma P-tau217 values below the lower detection limit of the assay were interpolated from the standard curve or, if this was not possible owing to the very low signal, the values were imputed to the lowest interpolated value. The main results excluding the values below the detection limit were similar and are shown in the eResults and eFigures 3 and 4 of the Supplement.

Two-sided *P* < .05 was considered statistically significant. All analyses were performed using SPSS, version 26 (IBM Corp) and R, version 3.6 (R Project for Statistical Computing).

## Results

### Participants

**Figure 1.**
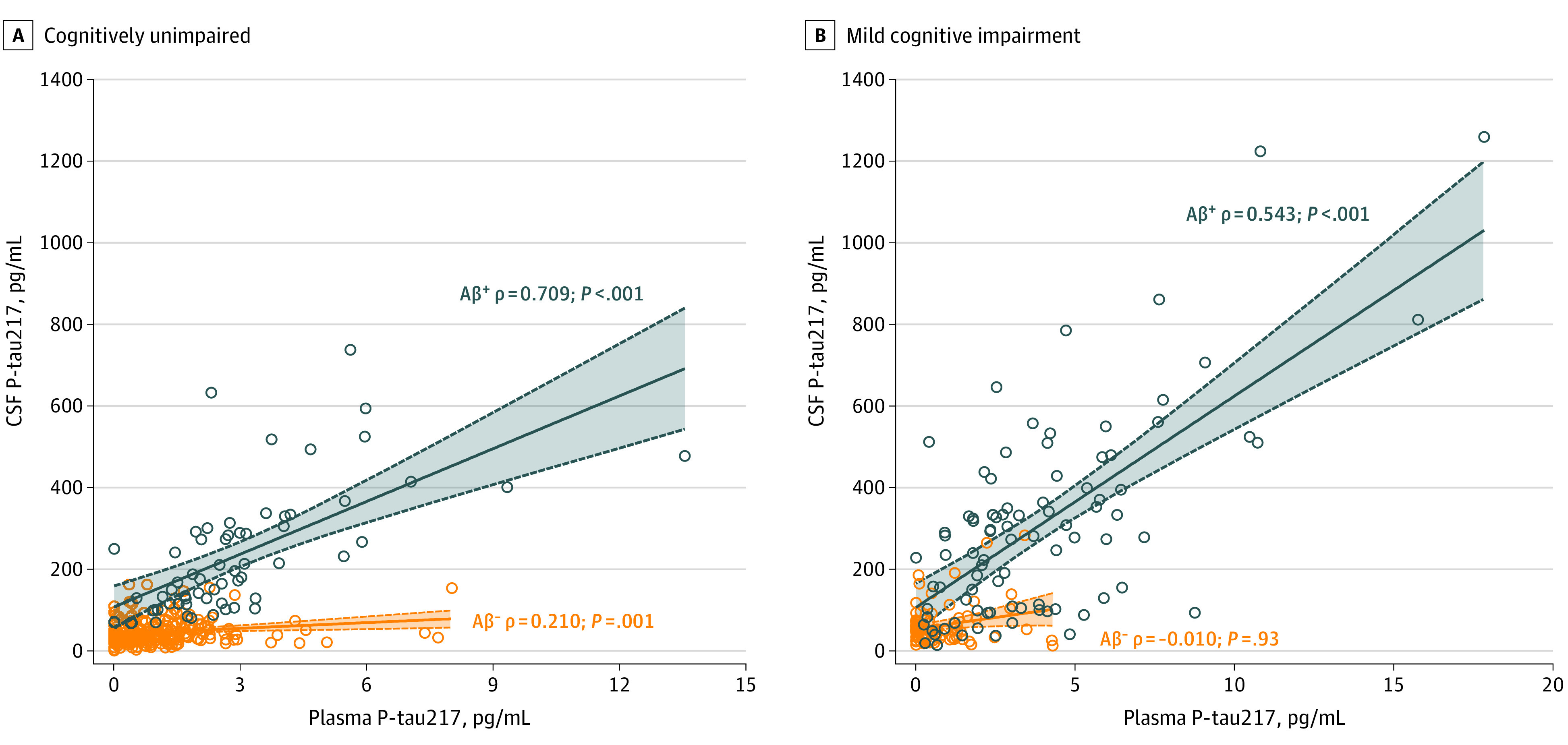
Association Between Plasma and Cerebrospinal Fluid (CSF) Tau Phosphorylated at Threonine 217 (P-tau 217) A and B, Association between plasma and CSF P-tau217 in cognitively unimpaired participants (amyloid-β positron emission tomography negative [Aβ-PET^–^], n = 252; Aβ-PET^+^, n = 61) (A) and participants with mild cognitive impairment (Aβ-PET^–^, n = 83; Aβ-PET^+^, n = 89) (B).

Of 490 participants, 251 (51.2%) were women and the mean (SD) age was 65.9 (13.1) years. The baseline demographic and clinical characteristics are summarized in the Table. Plasma P-tau217 correlated with CSF P-tau217 in Aβ-PET^+^ cognitively unimpaired participants (ρ = 0.709; *P* < .001) and in Aβ-PET^+^ participants with MCI (ρ = 0.543; *P* < .001) (Figure 1). Similar to CSF P-tau217, higher levels of plasma P-tau217 were associated with an increase in tau pathology in the brain measured using tau-PET (tau-PET^–^ in the entorhinal ROI, temporal meta-ROI and neocortical meta-ROI: median, 0.8 pg/mL; interquartile range [IQR], 0.3-1.7 pg/mL; tau-PET^+^ in the entorhinal ROI but not in the temporal meta-ROI: median, 3.1 pg/mL; IQR, 1.8-5.8 pg/mL; tau-PET^+^ in the temporal meta-ROI but not in the neocortical meta-ROI: median, 4.0 pg/mL; IQR, 2.3-5.7 pg/mL; tau-PET^+^ in the neocortical meta-ROI: median, 6.1 pg/mL; IQR, 5.0-10.6 pg/mL; eFigures 1 and 2 in the Supplement).

**Table.** Demographic and Clinical Characteristics of the Study Sample^a^

Characteristic	Cognitively unimpaired participants (n = 314)	Participants with mild cognitive impairment (n = 176)	*P* value
Age, y	64.7 (53.2-75.2)	72.2 (65.5-75.9)	<.001
Women, No. (%)	171 (54.5)	80 (45.5)	.06
Duration of education, y	12.3 (10.0-15.0)	12.0 (9.0-15.0)	.25
MMSE	29.0 (28.0-30.0)	27.0 (25.3-29.0)	<.001
*APOE* ε4 positivity, No. (%)	139 (44.3)	94 (53.4)	.06
Aβ-PET			
[^18^F]flutemetamol SUVR neocortical meta-ROI	0.47 (0.46-0.50)	0.55 (0.46-0.75)	<.001
Tau-PET			
[^18^F]RO948 SUVR, entorhinal ROI	1.11 (1.03-1.22)	1.22 (1.09-1.62)	<.001
[^18^F]RO948 SUVR, temporal meta-ROI^b^	1.15 (1.09-1.21)	1.20 (1.13-1.36)	<.001
[^18^F]RO948 SUVR, neocortical meta-ROI	1.05 (1.00-1.10)	1.06 (1.00-1.15)	.11
P-tau217 levels, pg/mL			
Plasma	0.96 (0.36-1.81)	1.53 (0.34-3.40)	<.001
CSF^c^	45.90 (28.31-88.43)	93.42 (46.30-288.96)	<.001

Abbreviations: Aβ, amyloid-β; *APOE*, apolipoprotein E; CSF, cerebrospinal fluid; [^18^F]flutemetamol, flutemetamol F 18; [^18^F]RO948, RO948 F 18; MMSE, Mini-Mental State Examination; PET, positron emission tomography; P-tau217, tau phosphorylated at threonine 217; ROI, region of interest; SUVR, standardized uptake value ratio.

^a^ Differences between the groups were tested using Mann-Whitney *U* and χ^2^ (sex and *APOE* gene) tests. Data are presented as median (interquartile range) unless otherwise specified.

^b^ [^18^F]RO948 SUVR data in the temporal meta-ROI were missing for 1 participant.

^c^ Cerebrospinal fluid P-tau217 data were missing for 5 participants.

### Plasma P-tau217 and Tau-PET Positivity in the Entorhinal Cortex of Cognitively Unimpaired Participants

**Figure 2.**
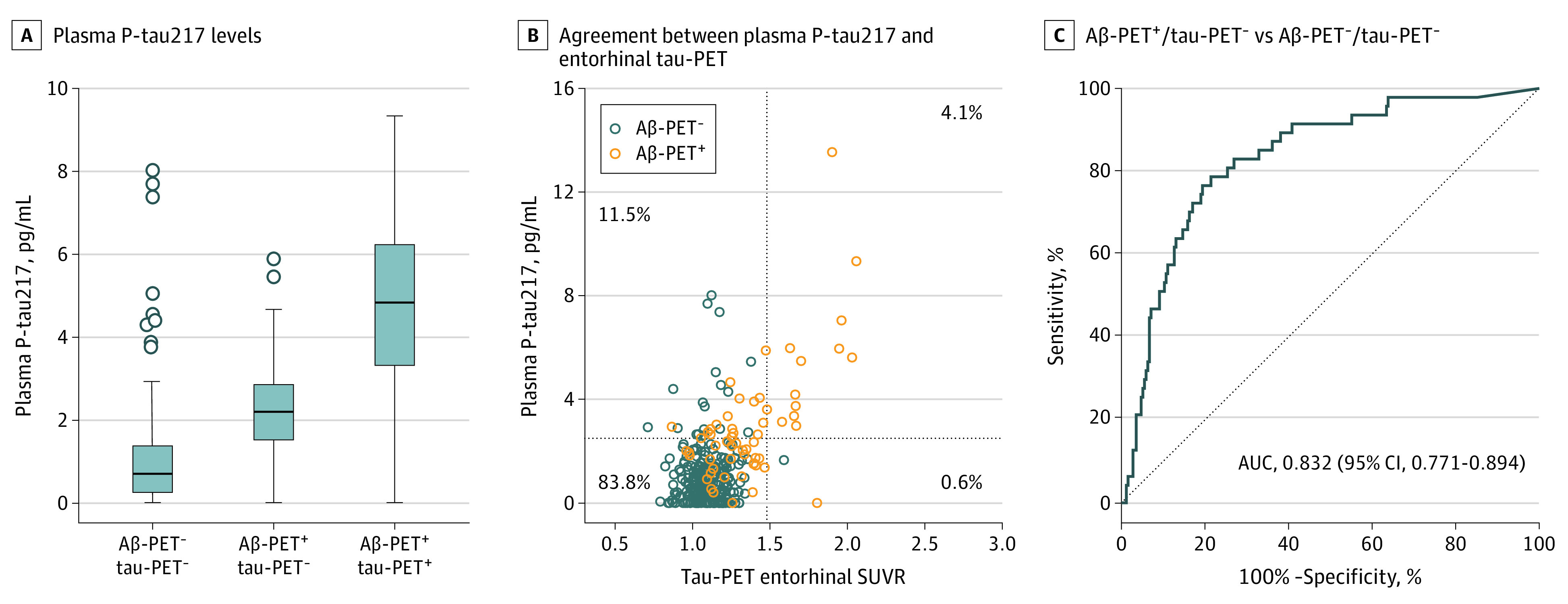
Plasma Tau Phosphorylated at Threonine 217 (P-tau217) and Tau–Positron Emission Tomography (PET) Positivity in the Entorhinal Cortex of Cognitively Unimpaired Participants A, Plasma P-tau217 levels in participants with amyloid-β (Aβ)–PET^–^/ tau-PET^–^ (n = 252), participants with Aβ-PET^+^/ tau-PET^–^(n = 47), and participants with Aβ-PET^+^/ tau-PET^+^ (n = 14) (*P* < .001 for Aβ-PET+/ tau-PET– vs Aβ-PET–/tau-PET– and Aβ-PET+/tau-PET+ vs Aβ-PET–/ tau-PET–). *P* values are from univariate general linear models adjusted for age and sex as described in the Methods section. Boxes indicate 25th to 75th percentiles, center lines indicate median, whiskers extend to the upper and lower adjacent values or the most extreme points within 1.5 × interquartile range of the 25th and 75th percentiles, and dots indicate outliers. B, Agreement between plasma P-tau217 and entorhinal tau-PET. The dotted lines represent cutoffs for plasma P-tau217 (2.5 pg/mL) and entorhinal tau-PET (standardized uptake value ratio [SUVR], 1.48). C, Receiver operating characteristic curve analysis for differentiating participants with Aβ-PET^+^/ tau-PET^–^ (n = 47) from those with Aβ-PET^–^/ tau-PET^–^ (n = 252). Cutoffs for plasma P-tau217, entorhinal tau-PET, and Aβ-PET (SUVR, 0.53) were determined as described in the Methods section. AUC indicates area under the curve.

When tau-PET status was defined based on the [^18^F]RO948 SUVR in the entorhinal ROI, of 314 cognitively unimpaired participants, 252 (80.3%) were classified as being Aβ-PET^–^/tau-PET^–^, 47 (15.0%) as being Aβ-PET^+^/tau-PET^–^, 14 (4.5%) as being Aβ-PET^+^/tau-PET^+^, and only 1 (0.3%) as being Aβ-PET^–^/tau-PET^+^. We found differences in plasma P-tau217 concentrations among the Aβ-PET^–^/tau-PET^–^, Aβ-PET^+^/tau-PET^–^, and Aβ-PET^+^/tau-PET^+^ groups (Figure 2A). Similar to CSF P-tau181 and CSF P-tau217, plasma P-tau217 levels were increased in Aβ-PET^+^/tau-PET^–^ cognitively unimpaired participants (median, 2.2 pg/mL; IQR, 1.5-2.9 pg/mL) compared with Aβ-PET^–^/tau-PET^–^ cognitively unimpaired participants (median, 0.7 pg/mL; IQR, 0.3-1.4 pg/mL) (Figure 2A); that is, the levels of plasma P-tau217 were increased in Aβ-positive cases even though tau-PET did not show evidence of paired helical filament–tau aggregates in the entorhinal cortex. Plasma P-tau217 accurately distinguished Aβ-PET^+^/tau-PET^–^ cognitively unimpaired participants from Aβ-PET^–^/tau-PET^–^ cognitively unimpaired participants with an area under the receiver operating characteristic curve of 0.832 (95% CI, 0.771-0.894) (Figure 2C) and 79% sensitivity and specificity (Youden index, 0.573).

There was a high agreement (87.9%) between binarized plasma P-tau217 and entorhinal tau-PET data. Most individuals with discordance were positive for P-tau217 and negative for tau-PET (36 of 38 [94.7%] were P-tau217^+^/tau-PET^–^, and 2 of 38 [5.3%] were P-tau217^–^/tau-PET^+^) (Figure 2B).

### Order of Change of Plasma P-tau217, CSF P-tau217, and Different Tau-PET Measures in Cognitively Unimpaired Participants and in Those With MCI 

**Figure 3.**
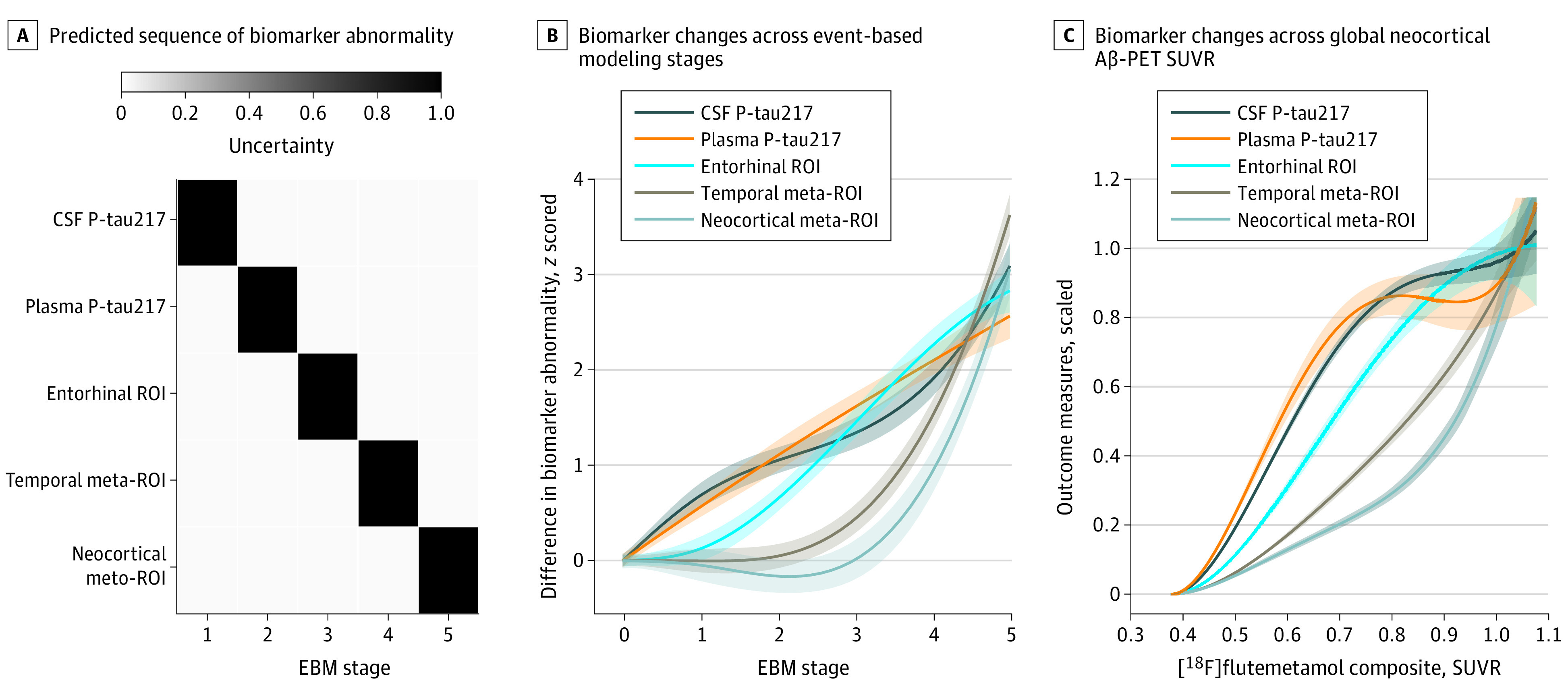
Order of Change in Plasma Tau Phosphorylated at Threonine 217 (P-tau217), Cerebrospinal Fluid (CSF) P-tau217, and Tau–Positron Emission Tomography (PET) Abnormality Order of change in plasma P-tau217, CSF P-tau217, and tau-PET abnormality in cognitively unimpaired participants and those with mild cognitive impairment (n = 484). A, Predicted sequence of biomarker abnormality from event-based modeling (EBM). Gray scale coding indicates uncertainty. B, Visualization of biomarker changes across EBM stages using nonlinear spline models. Uncertainties represent 95% CIs from the model-estimated variance-covariance matrix. C, Summary of biomarker changes in relation to global neocortical amyloid β (Aβ)–PET. All biomarkers are on a common scale ranging from 0 (baseline levels) to 1 (the mean levels in the top 10 percentiles). ROI, region of interest; and SUVR, standardized uptake value ratio.

In accordance with the aforementioned results, event-based modeling predicted that CSF and plasma P-tau217 changed before tau-PET measures, including the early tau accumulating entorhinal ROI (Figure 3A). Visualization using natural spline models suggested similarly early and steep increases in P-tau217 markers followed by tau-PET measures (Figure 3B).

In line with other findings of the present study, plasma and CSF P-tau217 levels were increased at lower Aβ-PET SUVR preceding the increase in tau-PET SUVR in the entorhinal ROI, followed by the increase in tau-PET SUVR in the temporal meta-ROI and neocortical meta-ROI (Figure 3C).

### Plasma P-tau217 Mediation of Aβ-PET and Tau-PET

**Figure 4.**
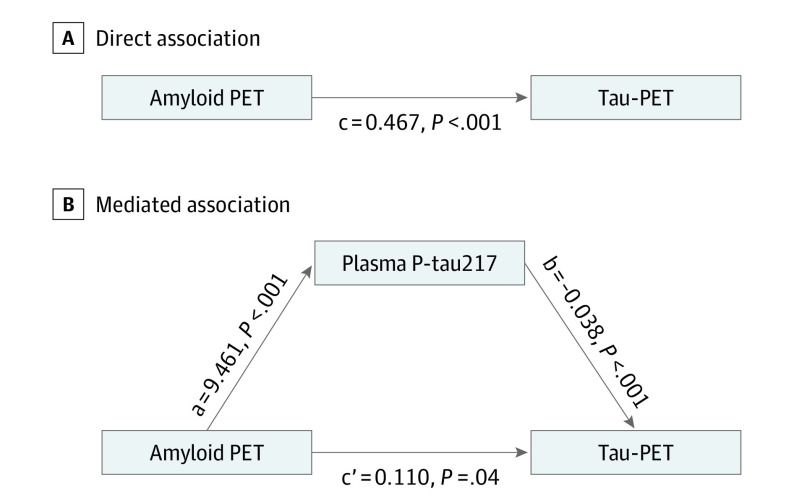
Mediated Effect of Plasma Tau Phosphorylated at Threonine 217 (P-tau217) on the Association of Amyloid-β (Aβ) Positron Emission Tomography (PET) With Tau-PET Mediated effect of plasma P-tau217 on the association of Aβ-PET with tau-PET in cognitively unimpaired participants and in those with mild cognitive impairment (n = 484). A, The direct association (*c*) of Aβ-PET with tau-PET. B, The mediated effect of plasma P-tau217 is designated *c-c*′. The remaining association of Aβ-PET with tau-PET after adjusting for plasma P-tau217 is designated *c*′. The direct association of Aβ-PET with plasma P-tau217 is *a*, and the association of plasma P-tau217 with tau-PET is *b* (*c-c*′ = 0.357; 95% CI, 0.281-0.435; 76.4%). This indicates that plasma P-tau217 mediated 76.4% of the effect of Aβ-PET on tau-PET.

In the mediation analysis including cognitively unimpaired participants and those with MCI (Figure 4), plasma P-tau217 significantly mediated the association between Aβ-PET and tau-PET to a large extent (partial mediation, 76.4%). However, there remained a direct smaller association (23.6%) of Aβ-PET with tau-PET.

### Plasma P-tau217 Levels and Longitudinal Changes in Entorhinal Tau-PET

A total of 111 individuals (72 [64.9%] cognitively unimpaired and 39 [35.1%] with MCI) who had normal tau-PET signal in the entorhinal cortex at baseline underwent 2 (n = 91) or 3 (n = 20) tau-PET scans (mean [SD] time between the first and the last scans, 1.6 [0.3] years; range 0.7-2.1 years). The yearly rate of increase in entorhinal tau-PET SUVR was higher in the group with high (>2.5 pg/mL) baseline levels of plasma P-tau217 compared with the group with low (≤2.5 pg/mL) baseline plasma P-tau217 levels (median SUVR, 0.029 [IQR, –0.006 to 0.041] vs –0.001 [IQR, –0.021 to 0.020]; Mann-Whitney *U*, *P* = .02); this corresponds to 2.2% increase per year from baseline in the P-tau217^+^ group.

## Discussion

In this study, plasma P-tau217 was increased in cognitively unimpaired participants with pathological Aβ-PET when the tau-PET signal in the entorhinal cortex was still normal and accurately differentiated between Aβ-PET^+^/tau-PET^–^ cognitively unimpaired participants and Aβ-PET^–^/ tau-PET^–^ cognitively unimpaired participants. There was a high agreement between plasma P-tau217 and tau-PET entorhinal ROI status (normal vs abnormal) among cognitively unimpaired participants, and the majority of individuals with discordance were positive for P-tau217 and negative for tau-PET. Event-based modeling of cross-sectional data predicted that plasma P-tau217 increased and became abnormal first followed by tau-PET measures in different brain regions linked to AD pathology. Study participants with normal baseline tau-PET and high baseline plasma P-tau217 had a higher longitudinal increase in tau-PET in the entorhinal cortex compared with those with low baseline plasma P-tau217.

Previous research has shown different patterns of changes in CSF P-tau and tau-PET measures across the AD continuum. Cerebrospinal fluid levels of P-tau increase in the earliest disease stages (ie, in asymptomatic individuals with CSF or PET evidence of abnormal Aβ accumulation) and appear to reach a plateau or even decrease in later symptomatic stages of AD.^14,24,25^ Tau-PET measures start to increase later in conjunction with brain atrophy and the appearance of cognitive symptoms and continue to increase with disease progression.^13,14,16,17^ Although high levels of plasma P-tau217 have already been reported in Aβ^+^ cognitively unimpaired participants compared with Aβ^–^ cognitively unimpaired participants,^12^ to our knowledge, here we showed for the first time that plasma P-tau217 was increased in Aβ-PET^+^ cognitively unimpaired participants before tau-PET positivity in the entorhinal cortex. Furthermore, we demonstrated that among cognitively unimpaired participants with normal entorhinal tau-PET, plasma P-tau217 accurately identified those who were Aβ-PET^+^ (with area under the receiver operating characteristics curve of 0.832 and sensitivity and specificity of 79%). These findings suggest that similar to CSF P-tau217, plasma P-tau217 might be a more useful biomarker than tau-PET in the earliest stages of AD (especially given the cost and accessibility of a blood test), but this needs to be further tested in longitudinal studies. Recent clinical trials^26,27^ of aducanumab in sporadic AD and gantenerumab in familial AD have shown that these anti-amyloid drugs reduce CSF levels of P-tau, indicating that they have downstream effects on tau metabolism. According to the present results, plasma P-tau217 could serve as a tool in clinical trials for (1) selection of individuals with preclinical AD who harbor early stage tau pathology before insoluble tau aggregates are prevalent and detectable by tau-PET and (2) monitoring target engagement of certain anti-tau drugs (given that these drugs do not interfere with the assay performance) and downstream pharmacodynamic effects on tau pathology by both anti-amyloid and anti-tau treatments.

The reasons for the discordance between CSF P-tau and tau-PET measures are not well understood. Some studies have shown that Aβ pathology in AD might trigger increased production, release, and phosphorylation of tau^28,29^ and that this likely happens early in the disease course because CSF levels of P-tau increase several years before tau aggregation is detectable by PET.^14^ In the present study, we used event-based modeling and cross-sectional data to predict the sequence of biomarker abnormality in cognitively unimpaired participants and in those with MCI. This modeling approach suggested that plasma P-tau217 became abnormal after CSF P-tau217 but before tau-PET in the entorhinal ROI, which was followed by temporal meta-ROI and then neocortical meta-ROI. Furthermore, nonlinear spline models estimating change in biomarkers by Aβ-PET load showed the same biomarker sequence: plasma and CSF P-tau217 levels were increased at lower Aβ-PET SUVR than tau-PET SUVR in the entorhinal ROI, temporal meta-ROI, and neocortical meta-ROI. Of note, among cognitively unimpaired participants and those with MCI with negative baseline tau-PET, high levels of plasma P-tau217 at baseline were associated with higher future increases in tau-PET SUVR in the entorhinal cortex, indicating that plasma P-tau217 may predict a subsequent increase in entorhinal tau-PET. In a previous study,^14^ tau-PET increases occurred only in individuals who also had increased CSF P-tau levels, and CSF P-tau mediated up to 80% of the association of Aβ-PET with tau-PET. On the basis of these findings, we proposed that Aβ pathology is associated with increased release and phosphorylation of tau (and consequently elevated levels of CSF P-tau), which later leads to accumulation of tau aggregates. Similarly, in the present study, plasma P-tau217 to a large extent mediated the association of Aβ-PET with tau-PET. These results suggest that Aβ-related changes in soluble tau metabolism in the early disease stages may be reflected not only in CSF but also in the plasma P-tau217 pool and thus further support plasma P-tau217 as an early biomarker of AD.

### Limitations

Limitations of the present study are the relatively small number of participants with longitudinal tau-PET scans, lack of longitudinal plasma P-tau217 data, and relatively young age of cognitively unimpaired participants. Future studies in large cohorts should investigate the dynamics of plasma P-tau217 and tau-PET changes over time in relation to Aβ positivity. The findings of the present study should also be validated for other tau-PET tracers. Another limitation is that plasma P-tau217 levels were below the detection limit of the assay for some of the cases. Thus, implementation of plasma P-tau217 as a biomarker of AD would benefit from the development of more sensitive assays suitable for detection of plasma P-tau217 at very low concentrations. A previous study^12^ using the same assay showed that 99% of the individuals with plasma P-tau217 values below the lower detection limit had normal tau-PET findings, and all plasma P-tau217 values were included in the main part of the present study. However, the results were similar when all data below the lower detection limit were excluded from the analysis (eFigures 3 and 4 and eResults in the Supplement).

## Conclusions

In this study, plasma levels of P-tau217 were increased in early preclinical AD, and the change preceded tau-PET positivity. High levels of plasma P-tau217 in people with normal tau-PET were associated with a higher future increase in tau-PET signal in the entorhinal ROI. These findings suggest that plasma P-tau217 is a promising biomarker of early AD that might be particularly useful for patient selection and as an outcome measure to monitor drug responses in clinical trials including individuals with preclinical AD.

## Notes

Supplement. **eMethods.** Participants and Analyses **eResults.** Sensitivity Analysis Excluding Plasma P-tau217 Data Below the Lower Detection Limit of the Assay **eFigure 1.** Voxel-Based Associations of Plasma P-tau217 and CSF P-tau217 With Tau-PET **eFigure 2.** Plasma P-tau217 and CSF P-tau217 Across the Braak ROI Groups **eFigure 3.** Plasma P-tau217 and Tau-PET Positivity in the Entorhinal Cortex of Participants Without Cognitive Impairment **eFigure 4.** Order of Change in Plasma P-tau217, CSF P-tau217 and Tau-PET Abnormality **eReferences**
