# Supplementary material for: Associations of Plasma Phospho-Tau217 Levels With Tau Positron Emission Tomography in Early Alzheimer Disease
Source: JAMA Neurol. 2020 Nov 9;78(2):1–8. doi: 10.1001/jamaneurol.2020.4201 (PMC7653537; doi:10.1001/jamaneurol.2020.4201)
Supplement: Supplement. — eMethods. Participants and Analyses eResults. Sensitivity Analysis Excluding Plasma P-tau217 Data Below the Lower Detection Limit of the Assay eFigure 1. Voxel-Based Associations of Plasma P-tau217 and CSF P-tau217 With Tau-PET eFigure 2. Plasma P-tau217 and CSF P-tau217 Across the Braak ROI Groups eFigure 3. Plasma P-tau217 and Tau-PET Positivity in the Entorhinal Cortex of Participants Without Cognitive Impairment eFigure 4. Order of Change in Plasma P-tau217, CSF P-tau217 and Tau-PET Abnormality eReferences [file jamaneurol-e204201-s001.pdf]

Supplementary Online Content

Janelidze S, Berron D, Smith R, et al. Associations of plasma phospho-tau217 levels with tau positron emission tomography in early Alzheimer Disease. *JAMA Neurol*. Published online November 9, 2020. doi:10.1001/jamaneurol.2020.4201

eMethods. Participants and Analyses

eResults. Sensitivity Analysis Excluding Plasma P-tau217 Data Below the Lower Detection Limit of the Assay

eFigure 1. Voxel-Based Associations of Plasma P-tau217 and CSF P-tau217 With Tau-PET

eFigure 2. Plasma P-tau217 and CSF P-tau217 Across the Braak ROI Groups

eFigure 3. Plasma P-tau217 and Tau-PET Positivity in the Entorhinal Cortex of Cognitively Unimpaired Participants (sensitivity analysis excluding plasma P-tau217 values below the detection limit)

eFigure 4. Order of Change in Plasma P-tau217, CSF P-tau217 and Tau-PET Abnormality (sensitivity analysis excluding plasma P-tau217 values below the detection limit)

eReferences

This supplementary material has been provided by the authors to give readers additional information about their work.

© 2020 Janelidze S et al. *JAMA Neurol.*

eMethods. Participants and Analyses

Study Participants

The study was approved by the Regional Ethics Committee in Lund, Sweden. All participants gave their informed consent to participate in the study. We included neurologically and cognitively healthy controls and patients with subjective cognitive decline (SCD) or MCI from the prospective and longitudinal Swedish BioFINDER-2 study who underwent both tau-PET and Aβ-PET imaging. Participants in the BioFINDER-2 study are recruited in southern Sweden (Skåne University Hospital and the Hospital of Ängelholm) as previously described.[1] The inclusion criteria for controls were i) ages 40-100 years; ii) absence of cognitive symptoms as assessed by a physician specialized in cognitive disorders; iii) MMSE score 27-30 (40-65 years of age) or 26-30 (66-100 years of age) at screening visit; iv) do not fulfill the criteria for mild or major neurocognitive disorder (MCI or dementia) according to DSM-5; and v) fluent in Swedish. The recruitment process of neurologically and cognitively healthy controls were designed to build two study populations with 50% *APOE* ε4 carriers in each. Inclusion criteria for patients with SCD or MCI were: i) Age 40-100 years; ii) referred to the memory clinics due to cognitive symptoms; iii) MMSE score of 24-30 points; iv) does not fulfill the criteria for any dementia (major neurocognitive disorder) according to DSM-5, v) fluent in Swedish. Exclusion criteria for both controls and patients were: i) significant unstable systemic illness that makes it difficult to participate in the study; ii) current significant alcohol or substance misuse; iii) refusing lumbar puncture, MRI or PET. Participants were classified as having MCI if they performed worse than -1.5 SD in any cognitive domain according to age and education stratified test norms. The neuropsychological battery covered the domains attention (Trail Making Test A and Symbol Digit Modalities Test), executive function (Trail Making B and A Quick Test of cognitive speed [AQT]), verbal ability (verbal fluency animals and the 15 word short version of the Boston Naming Test), memory (immediate and delayed recall from the Alzheimer’s Disease Assessment Scale [ADAS]), and visuospatial function (incomplete letters and cube analysis from the Visual Object and Space Perception battery [VOSP]). Those patients that were not classified as MCI were considered to have SCD. In accordance with the research framework by the National Institute on Aging-Alzheimer’s Association study patients with SCD and cognitively healthy individuals were included in the CU group.[2]

Plasma and Cerebrospinal Fluid (CSF) Sampling and Analysis

Concentrations of plasma P-tau217[3] and CSF P-tau217[4] were measured using Mesoscale Discovery (MSD) based immunoassays at Lilly Research Laboratories, IN, USA by technicians who were blinded to the clinical and imaging data. For plasma analysis, biotinylated-IBA493 was used as a capture antibody and SULFO-TAG-4G10-E2 (anti-Tau) as the detector. For CSF analysis, biotinylated-IBA413 was used as a capture antibody and a tau-specific antibody (LRL) as the detector. Plasma and CSF samples were diluted 1:2 and 1:8, respectively, in sample buffer containing heterophilic blocking reagent 1 at a concentration of 200 µg/ml (Scantibodies Inc). Both plasma and CSF assays were calibrated using a recombinant tau (4R2N) protein that was phosphorylated in vitro using a reaction with glycogen synthase kinase-3 and characterized by mass spectrometry.

For both plasma and CSF analysis, the sample was thawed on wet ice. Plasma samples were briefly vortexed, and centrifuged at 2,000 g for 10 min. MSD small-spot streptavidin-coated plates were blocked for 1 h at room temperature with 200 µl of 3% BSA in DPBS. The plates were then washed three times with 200 µl of wash buffer (PBS + 0.05% Tween 20), and 25 µl of biotinylated capture biotinylated capture antibodies (plasma assay: IBA493 at 0.5 µg/ml; CSF assay: IBA413 at 0.1 µg/ml) were added to the wells and incubated for 1 h at room temperature. The plates were again washed three times with 200 µl of wash buffer, and 50 µl of diluted calibrator or sample was added to each well and incubated for 2 h at room temperature. The plates were then washed three times with 200 µl of wash buffer, and 25 µl of SULFO-tagged detection antibody was added (plasma assay: 4G10-E2 at 0.05 µg/ml; CSF assay: LRL at 0.5 µg/ml and incubated for 1 h at room temperature. The plates were washed a final time with 200 µl of wash buffer and 150 µl of 2×MSD Read Buffer T with Surfactant was added to each plate and read on the MSD SQ120 within 10 min of read buffer addition. All plate incubations were performed with 650 rpm shaking on a plate shaker.

Tau and Aβ PET Imaging and Processing

Tau-PET images were acquired on digital GE Discovery MI scanners 70-90 min post injection of ~370 MBq [^{18}F]RO948. Aβ-PET imaging was performed using the same scanner 90-110 min after the injection of ~185 MBq [^{18}F]Flutemetamol. Standardized uptake value ratios (SUVR) were calculated using the inferior cerebellar cortex as reference region for [^{18}F]RO948[5] and the pons as reference region for [^{18}F]Flutemetamol. FreeSurfer (version 5.3) parcellation of the T1-weighted MRI scan was applied to the PET data transformed to participants' native T1 space to extract mean regional SUVR values for each participant in predefined regions of interest (ROI) affected by tau and Aβ pathology in AD.[6] These included Braak I-II ROI (entorhinal cortex), temporal meta-ROI or Braak III-IV (inferior and

© 2020 Janelidze S et al. *JAMA Neurol.*

middle temporal cortices, fusiform gyrus, parahippocampal cortex and amygdala) and neocortical meta-ROI or Braak V-VI (posterior cingulate gyrus, caudal anterior cingulate gyrus, rostral anterior cingulate gyrus, precuneus, inferior parietal lobule, superior parietal lobule, insula, supramarginal gyrus, lingual gyrus, superior temporal gyrus, medial orbitofrontal gyrus, rostral middle frontal gyrus, lateral orbitofrontal gyrus, caudal middle frontal gyrus, superior frontal gyrus, lateral occipital gyrus, precentral gyrus, postcentral gyrus and paracentral gyrus) for tau-PET[7] as well as neocortical meta-ROI (prefrontal, lateral temporal, parietal, anterior cingulate, and posterior cingulate/precuneus) for Aβ-PET.[8] Entorhinal ROI, temporal meta-ROI and neocortical meta-ROI tau-PET data was binarized using predefined cutoffs of 1.48, 1.36 and 1.35 SUVR, respectively.[9] Aβ-PET data was binarized using a cutoff derived from mixture modeling (0.53 SUVR). Out of 490 participants who underwent PET imaging, 66 had abnormally high tau-PET ligand retention in the entorhinal ROI and 152 had abnormally high neocortical Aβ-PET SUVR.

Statistical Analysis

To derive the sequence of biomarker abnormality, we used the event-based model as further described in Young et al.[10] In brief, the biomarkers in our study constitute a set of 5 biomarker transition 'events', where each corresponds to a biomarker becoming abnormal: two P-tau217 events (plasma and CSF) and three tau-PET events (entorhinal, temporal meta and neocortical meta-ROIs). The most likely ordering of events and the respective uncertainty was estimated using the EBM[10,11] where each biomarker is either treated as 'normal', i.e. non-pathological, or 'abnormal'. An 'event' represents the switch from normal to abnormal and our dataset contains measurements of each biomarker for each subject. The sequence that maximizes the data likelihood is the most likely ordering of the events. In addition to defining the most likely event sequence, the EBM allows to evaluate for any sequence to establish the relative likelihood of all sequences (by taking Markov chain Monte Carlo samples). This provides information about the uncertainty of the event ordering. The positional variance diagram[11] as shown in Figure 3A displays both the maximum likelihood sequence as well as its uncertainty by plotting the likelihood that each event appears in the respective position in the sequence. More details on the EBM and the calculation of uncertainties can be found in Young et al.[10] Furthermore, every participant was assigned a stage by the event-based modelling which was used to further visualize the evolvement of biomarker abnormality across stages using non-linear spline models (using natural splines). Uncertainties represent 95% confidence intervals from the model estimated variance-covariance matrix. All biomarker measures were z-standardized and normalized with respect to the mean of individuals at stage 0.
For voxel-based analyses between tau-PET and plasma and CSF P-tau217, we used multiple regression models adjusted for age and sex ($P<0.05$, familywise error rate corrected) as implemented in SPM-12 (https://www.fil.ion.ucl.ac.uk/spm/).

eResults. Sensitivity Analysis Excluding Plasma P-tau217 Data Below the Lower Detection Limit of the Assay

We verified the main findings excluding 161 study participants (151 Aβ-PET$^-$ and 10 Aβ-PET$^+$) with plasma P-tau217 levels below the detection limit of the assay (0.48 pg/mL). In cognitively unimpaired (CU) participants, plasma P-tau217 concentrations differed between the Aβ-PET$^-$/ tau-PET$^-$, Aβ-PET$^+$/ tau-PET$^-$, and Aβ-PET$^+$/ tau-PET$^+$ groups (eFigure 3A). Plasma P-tau217 levels were increased in CU Aβ-PET$^+$/ tau-PET$^-$ (median [IQR] 2.3 [1.7-2.9]) compared to CU Aβ-PET$^-$/ tau-PET$^-$ (median [IQR] 1.3 [0.8-1.8]) (eFigure 3A). There was a high agreement between binarized plasma P-tau217 and entorhinal tau-PET data (82.0% concordance). The majority of discordant cases were positive for P-tau217 and negative for tau-PET (97.3% [n=36] of discordant cases were P-tau217$^+$/tau-PET$^-$, and 2.7% [n=1] were Ptau217$^-$/tau-PET$^+$) (eFigure 3B). Event-based modelling in CU and patients with mild cognitive impairment (MCI) revealed that plasma P-tau217, similar to CSF P-tau217, became abnormal before tau-PET measures including the early tau accumulating entorhinal ROI (eFigures 4A and B).

© 2020 Janelidze S et al. *JAMA Neurol.*

eFigure 1. Voxel-Based Associations of Plasma P-tau217 and CSF P-tau217 With Tau-PET

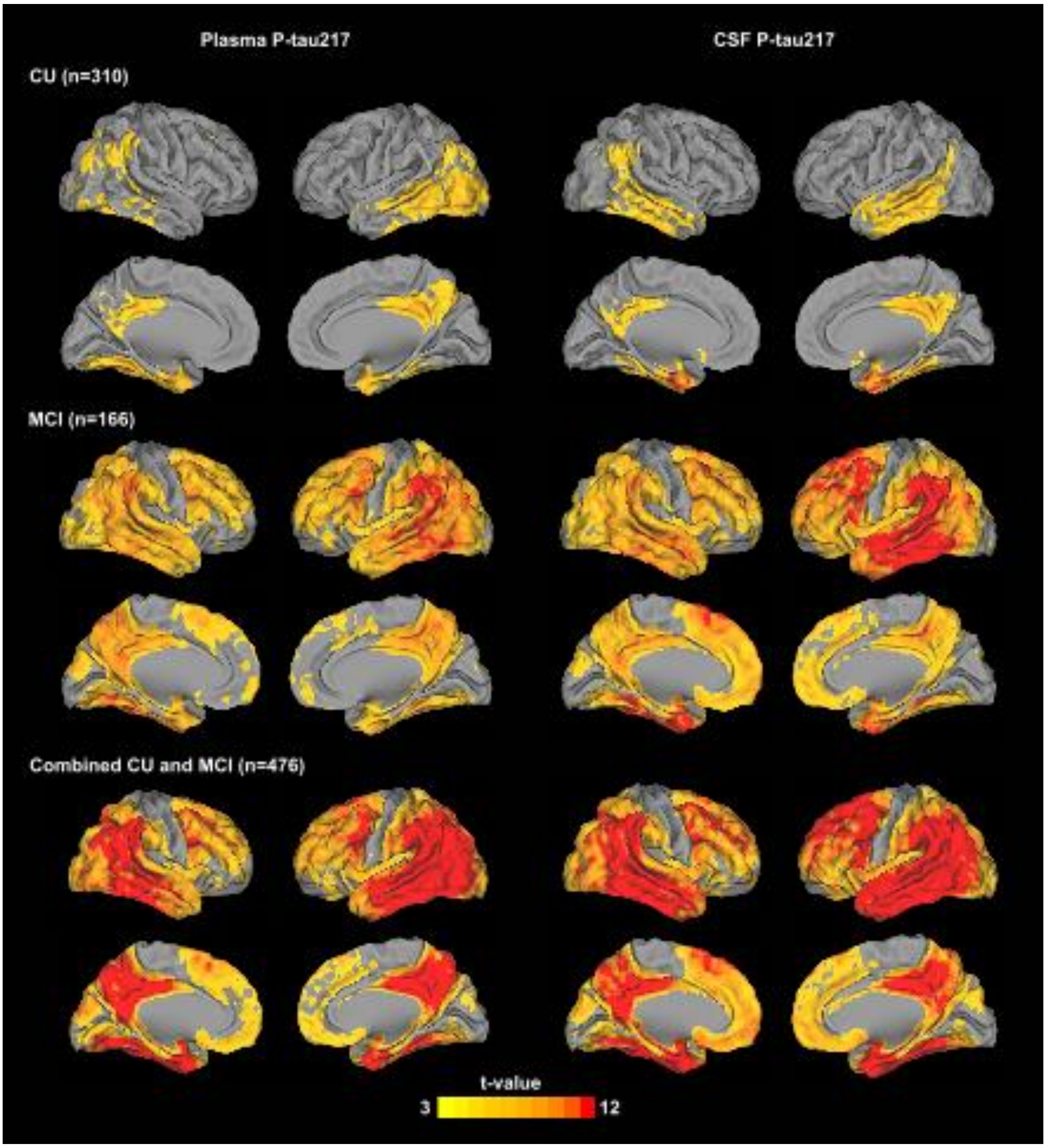

Voxel-based associations of plasma P-tau217 and CSF P-tau217 with tau-PET. Voxel-wise multiple regression analyses were adjusted for age and sex (P<0.05, familywise error rate corrected) as implemented in SPM-12 (https://www.fil.ion.ucl.ac.uk/spm/). Plasma P-tau217 and CSF P-tau217 data were both available for 485 individuals and nine subjects were excluded from the voxel-wise analyses due to problems with the normalization to MNI space. CSF = cerebrospinal fluid; CU = cognitively unimpaired; MCI, mild cognitive impairment; PET = positron emission tomography.

© 2020 Janelidze S et al. *JAMA Neurol.*

eFigure 2. Plasma P-tau217 and CSF P-tau217 Across the Braak ROI Groups

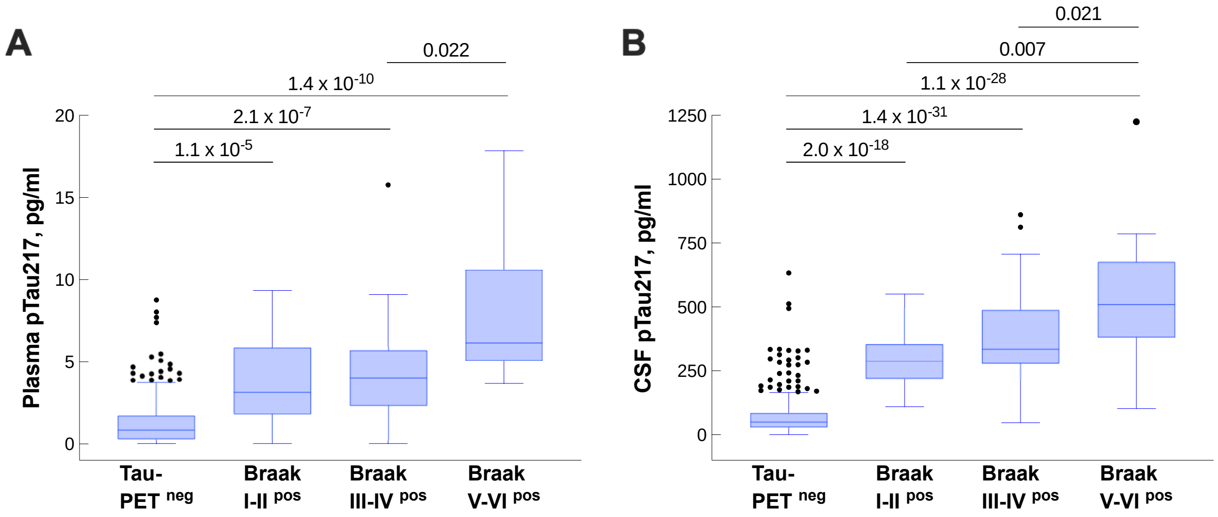

Plasma P-tau217 and CSF P-tau217 across the Braak ROI groups. Plasma (**A**) and CSF (**B**) concentrations of P-tau217 in individuals without significantly elevated Tau PET measurements in Braak I-II ROI, Braak III-IV ROI and Braak V-VI ROI (Tau PET negative), and those with significantly elevated measurements in one or more of these ROIs, including i) Braak I-II (but not III-VI), ii) Braak III-IV (but not V-VI) or iii) Braak V-VI. Data are from a subcohort of 485 individuals (313 CU and 172 MCI) where both plasma and CSF P-tau217 measures were all available. Tau data were binarized based on predefined cutoffs as described in eMethods. P-values are from univariate general linear models adjusted for age and sex as described in the methods.
CSF = cerebrospinal fluid; neg = negative; CU = cognitively unimpaired; MCI, mild cognitive impairment; PET = positron emission tomography ; pos = positive; ROI = region of interest.

eFigure 3. Plasma P-tau217 and Tau-PET Positivity in the Entorhinal Cortex of Participants Without Cognitive Impairment

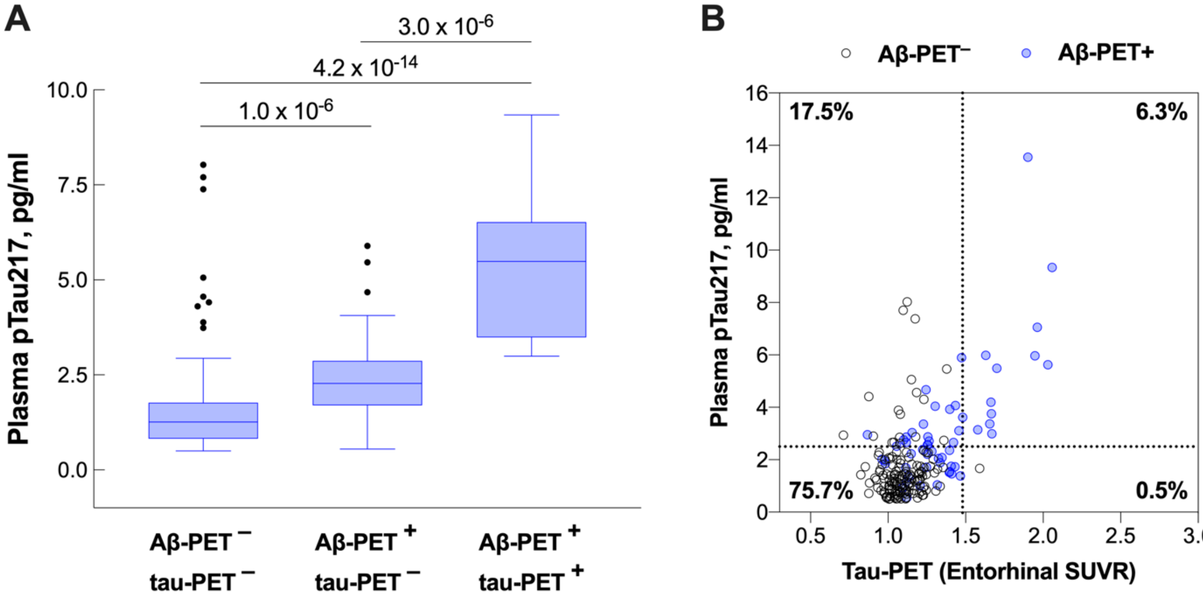

Plasma P-tau217 and tau-PET positivity in the entorhinal cortex of CU participants (sensitivity analysis excluding plasma P-tau217 values below the detection limit). **(a)** Plasma P-tau217 levels in the Aβ-PET$^-$/ tau-PET$^-$ (n=148), Aβ-PET$^+$/ tau-PET$^-$(n=44), and Aβ-PET$^+$/ tau-PET$^+$ (n=13). P-values are from univariate general linear models adjusted for age and sex as described in the methods. (**b**) Agreement between plasma P-tau217 and entorhinal tau-PET. The dotted lines represent cutoffs for plasma P-tau217 (2.5pg/ml) and entorhinal tau-PET (1.48 SUVR). Cutoffs for plasma P-tau217 (2.5 pg/ml), entorhinal tau-PET (1.48 SUVR) and Aβ-PET (0.53 SUVR) were determined as described in the methods.

© 2020 Janelidze S et al. *JAMA Neurol.*

AUC = area under the curve; Aβ = Amyloid-β; CI = confidence interval; PET = positron emission tomography; ROC = receiver operating characteristic; SUVR = standardized uptake value ratio.

eFigure 4. Order of Change in Plasma P-tau217, CSF P-tau217 and Tau-PET Abnormality

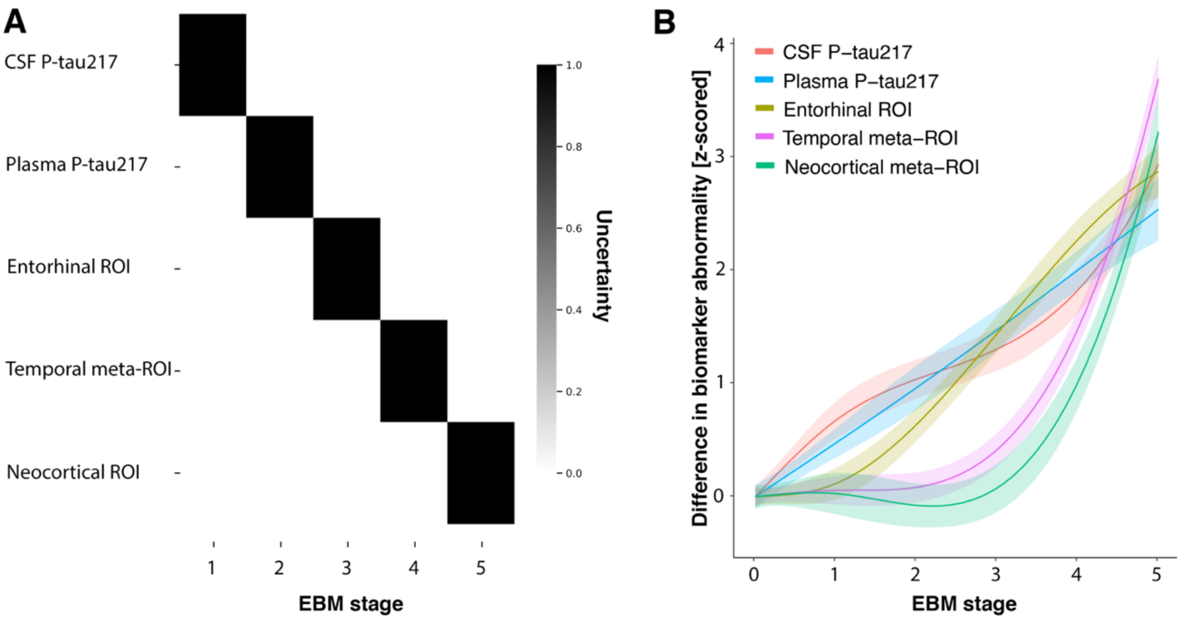

Order of change in plasma P-tau217, CSF P-tau217 and tau-PET abnormality (sensitivity analysis excluding plasma P-tau217 values below the detection limit). (**a**) Predicted sequence of biomarker abnormality from event-based modelling. Grey scale coding indicates uncertainty. (**b**) Visualization of biomarker changes across event-based modelling stages using non-linear spline models.
CSF = cerebrospinal fluid; EBM = event-based modelling; ROI = region of interest; SUVR = standardized uptake value ratio.

eReferences

1. Palmqvist S, Janelidze S, Quiroz YT, et al. Discriminative accuracy of plasma phospho-tau 217 for Alzheimer disease vs other neurodegenerative disorders. 2020; In press.
2. Jack CR, Jr., Bennett DA, Blennow K, et al. NIA-AA Research Framework: Toward a biological definition of Alzheimer's disease. *Alzheimers Dement.* 2018;14(4):535-562.
3. Janelidze S, Mattsson N, Palmqvist S, et al. Plasma P-tau181 in Alzheimer’s disease: relationship to other biomarkers, differential diagnosis, neuropathology and longitudinal progression to Alzheimer’s dementia. *Nature Medicine.* 2020;26(3):379-386.
4. Janelidze S, Stomrud E, Smith R, et al. Cerebrospinal fluid p-tau217 performs better than p-tau181 as a biomarker of Alzheimer's disease. *Nat Commun.* 2020;11(1):1683.

© 2020 Janelidze S et al. *JAMA Neurol.*

5. Baker SL, Maass A, Jagust WJ. Considerations and code for partial volume correcting [(18)F]-AV-1451 tau PET data. *Data Brief.* 2017;15:648-657.

6. Braak H, Braak E. Neuropathological stageing of Alzheimer-related changes. *Acta Neuropathol.* 1991;82(4):239-259.

7. Ossenkoppele R, Rabinovici GD, Smith R, et al. Discriminative Accuracy of [18F]flortaucipir Positron Emission Tomography for Alzheimer Disease vs Other Neurodegenerative Disorders. *JAMA.* 2018;320(11):1151-1162.

8. Lundqvist R, Lilja J, Thomas BA, et al. Implementation and validation of an adaptive template registration method for 18F-flutemetamol imaging data. *J Nucl Med.* 2013;54(8):1472-1478.

9. Leuzy A, Smith R, Ossenkoppele R, et al. Diagnostic Performance of RO948 F 18 Tau Positron Emission Tomography in the Differentiation of Alzheimer Disease From Other Neurodegenerative Disorders. *JAMA Neurol.* 2020.

10. Young AL, Oxtoby NP, Daga P, et al. A data-driven model of biomarker changes in sporadic Alzheimer's disease. *Brain.* 2014;137(Pt 9):2564-2577.

11. Fonteijn HM, Modat M, Clarkson MJ, et al. An event-based model for disease progression and its application in familial Alzheimer's disease and Huntington's disease. *Neuroimage.* 2012;60(3):1880-1889.

© 2020 Janelidze S et al. *JAMA Neurol.*
